# Ossification of the Arachnoid

**Published:** 1849-06

**Authors:** 


*Ossification of the Arachnoid.*—At the Pathological Society of London, Dr. Brinton exhibited a specimen of ossification of the arachnoid, taken from a patient who had died of some general disease, no suspicion of any affection of the meninges having been entertained during life.

It consisted of very numerous and small plates of ossific matter, lying immediately beneath the visceral layer of arachnoid. Their outer surface was smooth and covered by the serous membrane. Their inner surface rough and minutely mammillated; their little projections just visible with the naked eye, looking towards and into the sub-arachnoid space. The size of the plates varied, on an average, about one-eighth to one-fourth of an inch.

The microscope shewed them to consist of numerous conical processes, projecting from a flat stratum of bone. Rarely their shape was fungiform. Their several appearances somewhat resembled the pharyngeal teeth of some snails. In structure they consisted of canaliculi and lacunæ, without Haversian canals.—*Ibid.*

*On the existence of Free Carbon in the Human Body.* By JAMES PAXTON, M. D., of Rugby.—For some time past I have been occupied in collecting and arranging pathological evidence relating to the modes of production, the forms of retention, and the remarkable extrication of uncombined carbon in various parts of the human body. There are not wanting instances of the deposition of free carbon very manifest during life, but it is more frequently observed in post-mortem inspections, and more especially invading the pulmonary tissue. The fruits of these investigations I have much pleasure in placing before this meeting of the Association.

It is a curious fact, that the only elementary substance as yet detected in organized bodies, chemically uncombined, is carbon. There is no part of the animal system in which it is so frequently met with as in the bronchial glands. The true cause of their blackness, I believe, was first demonstrated by Dr. Pearson.

Whenever there are pulmonary lesions of a lengthened duration, or any mechanical interruptions to the respiratory functions, these glands become the repositories of carbon; the effect of which is to produce a degree of enlargement and induration. I have a specimen of carbonaceous bronchial gland enormously increased in its dimensions, and of an almost scirrhous density, with the pneumogastric nerve adherent to it. It is not unfrequently the case that the bronchial glands are so loaded with carbon that they are of an uniform inky colour. The extent of black discolouration bears some relation to the age of the person, to the state of the lung, and to the nature of the air breathed, particularly to the quantity of carbon it contained. The bronchial glands are always proportionably larger and blacker in those who are affected by chronic disease of the lungs. In persons advanced in life there is a disposition to carbonaceous retentions, more especially in the lungs; it indicates the period of senile atrophy at which they have arrived. A certain amount of black lines in the interlobular tissues, a gray or black web in the interior, occupying a greater or less space, with specks of various dimensions and granules of the same appearance, constantly characterize the lungs of the aged. A chemical analysis by Guillot has proved this black matter to be exclusively carbon in an exceedingly minute state of division. When this carbonaceous matter is blended together with the animal fluids it becomes a pigmentum nigrum, corresponding to the secretion of the choroid membrane, and to the ink of the cuttle-fish. The colouring matter of all these substances is animal charcoal, identical with the animal charcoal artificially produced in its indestructible properties, und remarkable in its resistance to the action of the strongest nitric and hydrochloric acids or the caustic alkalies.

Here I wish to be understood that the carbonof the lungs in the aged is not always derived from the inspiration of impure air, but that it appears frequently, at least, to depend on insufficient combustion.

Chronic pneumonia, whether simple or accompanied by tuberculous accretions, as a consequent affection, occasions accumulations of carbon, which are unequally insinuated in the pulmonary textures. It may be collected in large black masses, or it may be scattered in smaller isolated spots; at other times we find the black spots irregularly arranged in clusters. From such specimens we shall be able to form some tolerable conception of the results of chronic inflammation in the production of carbonaceous deposits. Hence vascular congestion has encroached on the air-vessels, and obliterated much of the areolæ, with even the ultimate ramifications of the bronchi. The air and blood are now no longer in contact, the extrication of carbonic acid ceases, and free carbon is detained in the lungs. We often observe large cavities at the apex lined by a carbonaceous pigment. During life these cavities communicated with the bronchi, therefore with the attendant cough was gray expectoration proceeding from a mixture of this substance with the mucous and suppurative secretions.

Tuberculosis or true phthisis pulmonalis, as it advances to a fatal issue, destroys the vesicular and tubular structures, consequently the aerial spaces become more and more limited, and in proportion to the extension of the disease, the chemical changes required in respiration are equally limited. If the disease is protracted, similar depositions, from the causes just mentioned, take place; sometimes we perceive them centrally in the tubercle, but usually the carbonaceous depositions circumscribe the tubercle. The effect of such black infiltration is induration of the part, with shrinking and corrugations. There are, however, cases of carbonaceous lungs in which there is no obvious alteration in form, the carbon being simply diffused through a congestive organization.

It is not my intention to review every known phenomenon relating to the retention of carbon, but merely to glance at the fact of its being found in various parts of the animal system. We feel little surprise at the discovery of carbon in the organ destined to decarbonize the blood; but we are rather astonished at its appearance in the organs remote from the lungs. I wish especially to draw attention to the consequences of carbonaceous affections of the lungs, particularly as there is a vague notion referred to by Hasse, that black deposit (denominated pseudo-melanosis), is not only innoxious, but actually opposes a barrier to the encroachments of tubercles, and is "the almost unfailing concomitant of reparation of pulmonary disease." Another admirable pathologist has asserted "black pulmonary matter to be altogether compatible with perfect health." Such views, hovever, are unsupported by the cases I have examined. On the contrary, I regard melanotic stains as marking a stage of degeneration, in which the vascular and aërial apparatus is most seriously involved. So far as uncombined carbon invades the lungs, in the same proportion are the circulation and the respiration impeded. We may, therefore, consider productions of black matter as no other than an unmixed evil. When added to turbercular mischief, it only increases the obstacles to the maintenance of the vital and chemical functions of the lungs, by farther abridging the areolar surface, and by obstructing the escape of certain normal and abnormal secretions which might happen to be the attendants on the carbonaceous diathesis.

To what cause are such retentions of carbon to be attributed? Brockman thought it " an effort of nature to relieve the circulation of a surcharge of carbon." Hensinger, I imagine, approaches nearly to the correct theory : namely, a deficient elimination of carbon, and particularly of carbonic acid, assigning this reason for the opinion, " carbon being principally found in organs which ought to be the natural outlet of this element."

In the cases I have studied, the retention of carbon arose under the following circumstances:—1st. During the reduction of animal power by lengthened years, when the pulmonary organs seem unequal to the task of expelling carbonic acid. 2d. Where the existence of disease prevents both the reception of oxygen and the escape of carbon from the lungs. 3d. Where the individual lived in a situation in which we might suppose there was insufficient oxygen in the atmosphere he breathed. It is not unreasonable to assume that every one of these causes, or a combination of them, may be sufficient to account for the retention of carbon in its passage through the vessels.

The next class of cases to which I shall advert are those in which carbonic acid, under certain morbid conditions, is decomposed on the surface, the carbon uncombined remaining on the skin. The extrication of carbon by perspiration is easily demonstrated by the colour of the linen when worn for too long a time. Free Carbon is also deposited on the epidermes of old persons, particularly under the nails, and " where free access of oxygen is impeded; for example, in the axilla, in the soles of the feet."

The most extraordinary instance of the production of carbon I have ever witnessed is that of a young lady, who having been in ill health for some years, from an affection of the heart, has now exudations of pure carbon on the skin round the orbits and about the mouth. It is washed off in the morning, but it reappears in the evening. When wiped away with a napkin, we are immediately struck by the black sooty appearance. In a word, this exudation has all the properties of the smoke collected from the flame of a lamp.

A similar case is recorded in the Medico-Chirurgical Transactions, of transpiration of carbon from the forehead. There is, therefore, competent authority for asserting that vital chemistry is capable of reducing one of the component parts of the body to its original element.

The series of cases just mentioned are entirely to be ascribed to an internal origin; the remote cause is organic, but the immediate cause is chemical, and constitutes a train of features attendant on the diversified diseases which interfere with the economy of respiration.

The last series of carbonaceous affections which I shall have occasion to notice are those in which the introduction of carbon into the lungs is from external and incidental causes. I allude to the inhalation and absorption of molecules of carbon suspended in the air in the form of smoke. In manufacturing towns, the ordinary carbonaceous lung is very common. The Professor of Medicine in Queen's College, Birmingham, has " never examined the body of an artizan, who was exposed by his occupation to the respiring of an atmosphere containing carbonaceous matter, without finding his lungs more or less blackened by it." The effect of this during life is to interpose mechanical impediments to the functions of the lungs, in addition to the irritation excited by the presence of extraneous matter.

One species of this series of carbonaceous lung is peculiar to those who labour in coal mines. There is a specimen in the collection of the Royal Medical Society of Edinburgh, showing the miserable condition of lung to which coal miner's are liable. There may be seen some fine jet black lungs in the Museum of the College of Surgeons in the same city. Just imagine the lungs carved out of a piece of coal, and you have a perfect idea of these specimens. I have in my possession a portion of miner's lung, beautifully spotted, given to me by Dr. Peacock. In describing the colour, it would be obvious to any one who would do me the favour to inspect it, that I have not exaggerated the description when I say that the whole of the pulmonary tissue is penetrated by minute atoms of carbon. Dr. Ayres did me the favor to examine it, and assures me that it contains a large portion of carbonaceous matter.

Perhaps there has been enough produced to prove that great damage is sustained by the pneumonic system, when, from whatever cause, carbonic acid is decomposed instead of being thrown off by expiration and the secretions. We ought to be no longer under the erroneous impression that the retention of uncombined carbon has any other than ill consequences. I have also endeavored to exhibit the equally injurious effects of breathing an atmosphere impregnated with smoke or coal-dust. To follow up the inquiry and to multiply instances would not be difficult, but does not fall within the compass of the present communication. The latter division of my subject is still under the deliberation of the government, and it is a subject on which all ranks would do well to reflect. If, in addition to much that has been lately published on these subjects, I have brought before you some direct evidence of the nature and consequences of carbonaceous affections, I have obtained my object. Philanthropists and philosophers have pointed out preventives if not remedies.

In the pathological division of the Museum of the College of Surgeons, in London, the preparations numbered 1,462, 1,477, 1,648, 1,798 A, may be referred to as fine specimens of several states of the lungs here described.—*Trans. of the Prov. Med. and Surg. Assoc.*

